# The American Agriculturist

**Published:** 1879-11

**Authors:** 


*The American Agriculturist.*—We advise all our readers, whether they own a foot of land or not, to supply themselves with that treasure of useful, practical, reliable information, the *American Agriculturist*, so named because started 38 years ago as a rural journal, but now enlarged to embrace a great variety of most useful reading for the Household, children included, for the Garden, as well as the Farm—for all classes. Each volume gives some 800 original Engravings, with descriptions of labor-saving and labor-helping contrivances, of plants, fruits, flowers, animals, etc., including many large and pleasing, as well as instructive, pictures for young and old. The constant, systematic exposures of Humbugs and Swindling Schemes by the *Agriculturist* are of great value to every one, and will save to most persons many times its cost. Altogether, it is one of the most valuable, as well as cheapest, Journals any where to be found. The cost is only $1.50 a year, or 4 copies for $5. Single numbers 15 cents. Subscribe at once for 1880, and receive the rest of this year free, or send 3-cent stamp for postage on a specimen copy. Address Orange Judd Company, Publishers, 245 Broadway, New York.

